# Intelligence and eeg measures of information flow: efficiency and homeostatic neuroplasticity

**DOI:** 10.1038/srep38890

**Published:** 2016-12-20

**Authors:** R. W. Thatcher, E. Palmero-Soler, D. M. North, C. J. Biver

**Affiliations:** 1 EEG and NeuroImaging Laboratory, Applied Neuroscience Research Institute. St. Petersburg, Fl, USA.

## Abstract

The purpose of this study was to explore the relationship between the magnitude of EEG information flow and intelligence. The electroencephalogram (EEG) was recorded from 19 scalp locations from 371 subjects ranging in age from 5 years to 17.6 years. The Wechler Intelligence Scale for Children (WISC-R) was administered for individuals between 5 years of age and 16 years and the Weschler Adult Intelligence Scale revised (WAIS-R) was administered to subjects older than 16 years to estimate I.Q. The phase slope index estimated the magnitude of information flow between all electrode combinations for difference frequency bands. Discriminant analyses were performed between high I.Q. (>120) and low I.Q. groups (<90). The magnitude of information flow was inversely related to I.Q. especially in the alpha and beta frequency bands. Long distance inter-electrode distances exhibited greater information flow than short inter-electrode distances. Frontal-parietal correlations were the most significant. It is concluded that higher I.Q. is related to increased efficiency of local information processing and reduced long distance compensatory dynamics that supports a small-world model of intelligence.

There are three main types of brain connectivity. One is structural connectivity as measured by structural MRI and diffusion tensor imaging. This level of connectivity is the same whether one is alive or shortly after death and represents the essential structural infra-structure of the brain. The second is functional connectivity as measured by EEG coherence and fMRI correlations between brain regions. This level measures the temporal correlation between two or more brain regions and indicates functional activity shared by the correlated regions. The third level is called effective connectivity which is a measure of the magnitude and direction of information flow between two or more connected brain regions^1^,^2^,^3^. By analogy structural connectivity is like the street connecting a parking lot to a sports stadium, functional connectivity is the correlation between changes in the two locations and effective connectivity measures the direction and magnitude of the flow of people that travel between the two locations.

Two related and commonly reported models of intelligence emphasize efficiency of information processing between the frontal lobes and the parietal lobes. EEG network studies have argued that increased complexity and increased neural efficiency are positively related to intelligence^4^,^5^,^6^,^7^,^8^,^9^,^10^,^11^,^12^,^13^. Studies by Haier *et al*.^14^ demonstrated greater gray matter volume in frontal and parietal regions in high I.Q. children and Langeslag *et al*.^15^ reported increased functional connectivity with BOLD fMRI in high I.Q. children.

A resolution of a specialization model of intelligence with emphasis on the frontal-parietal lobes and global efficient allocation of resources in a small-world model of the brain was provided by Thatcher *et al*.^10^,^11^ who showed both increased efficiency in global network dynamics as well as reduced phase differences and long phase shift durations in frontal-parietal relations positively correlated with I.Q. Langer *et al*.^12^ further demonstrated both increased small-world global connectivity as well increased hub order of the parietal lobes in high I.Q. subjects.

As mentioned previously, both structural and functional connectivity between frontal and parietal regions are positively correlated with higher I.Q. and both structural and functional connectivity measures indicate a general increased efficiency of global dynamics positively correlated with higher I.Q. For example, frontal-parietal EEG phase differences were shorter in higher I.Q. subjects^10^ and EEG phase lock durations were shorter in higher I.Q. subjects^11^. An added factor in understanding the nature of intelligence is the relationship between short distance connections vs long distance connections in complex networks. For example, in small-world models increased efficiency is related to increased differentiation or localization and minimization of long distance connections. Consistent with the global efficiency small-world models are studies showing weak long distance functional connectivity correlated with higher intelligence^8^. A complementary finding are correlations with higher intelligence in short distance EEG electrode combinations using EEG phase reset which is also consistent with a small-world model where reduced long distance connectivity and increased short distance connectivity are correlated with higher intelligence^11^. The EEG studies are also consistent with Graph theoretical models of intelligence using structural MRI. For example, van den Heuvel *et al*.^16^ and Li *et al*.^17^ found that higher I.Q. negatively correlated with path length and path length is inversely proportional to network efficiency. Thus, both structural connectivity and functional connectivity measures demonstrate a positive correlation between I.Q. and the efficiency of information processing in networks of the brain.

Previous studies from this laboratory on the relationship between intelligence and absolute power, coherence, amplitude asymmetry and phase differences have shown that phase and coherence produce stronger correlations to I.Q. than absolute power^9^. LORETA source correlations and I.Q., although weaker than coherence and phase, indicate specific timing relationships between 3-dimensional current sources and information processing^10^. A study of the relationship between EEG phase shift duration (“unstable phase dynamics”) and phase lock duration (“stability”) and human intelligence reported a positive relationship between the efficiency of information processing by phase shift and phase lock duration and intelligence^11^. An interesting finding in the phase reset and intelligence study was that primarily short interelectrode distance was correlated with intelligence and not long interelectrode distances. This indicated that intelligence and efficiency is related to the recruitment of local groups of neurons^11^. However, there have been no studies on the relationship between effective connectivity or the magnitude of information flow and intelligence which may yield information about the more global aspects of efficiency. Therefore, the purpose of the present study is to investigate the relationship between human EEG measures of effective connectivity using the phase slope index and neuropsychological measures of intelligence in the same subjects as studied in Thatcher *et al*.^9^,^10^,^11^,^18^. The phase slope index (PSI) is a measure of effective connectivity that estimates the magnitude and direction of information flow in the EEG^1^,^2^,^19^.

## Methods

### Subjects

A total population of 1,015 rural and urban children ranging in age from 2 months to 17.54 years of age were recruited as part of a Department of Agricultural study of the relationship between nutrition and brain development and this is why no adults beyond the age of 17.54 were included in this study^20^,^21^,^22^. The study was approved by a University of Maryland Institutional Review Board (IRB) and informed consent was obtained from the parents of all the subjects in this study. All methods were performed in accordance with the relevant guidelines and regulations. Two data acquisition centers were established, one at the rural University of Maryland Eastern Shore campus and one at the urban campus of the University of Maryland School of Medicine in Baltimore, Maryland. Identical data acquisition systems were built and calibrated, a staff was trained using uniform procedures and a clinical and psychometric protocol were utilized in the recruitment of subjects.

### Inclusion/Exclusion Criteria

From the total of 1,015 subjects, 371 subjects ranging in age from 5 years to 17.54 years were selected. a neurological history questionnaire given to the child’s parents and/or filled out by each subject, 2-psychometric evaluation of I.Q., and/or school achievement, 3- for children the teacher 9 and class room performance as determined by school grades and teacher reports and presence of environmental toxins such as lead or cadmium. A Neurological questionnaire was obtained from all of the adult subjects >18 years of age and those in which information was available about a history of problems as an adult were excluded. The inclusion criteria were: 1- no history of neurological disorders such as epilepsy, head injuries and reported normal development and successful school performance, 2- A Full Scale I.Q. >70; 3- WRAT School Achievement Scores >89 on at least two subtests (i.e., reading, spelling, arithmetic) or demonstrated success in these subjects and 4- A grade point average of ‘C’ or better in the major academic classes (e.g., English, mathematics, science, social studies and history).

### Demographic Characteristics

The subjects were made up of 58.9% males, 41.1% females, 71.4% Caucasian, 24.2% African American and 3.2% oriental. Socioeconomic status (SES) was measured by the Hollingshead four factor scale. Time of day was randomized and counter-balanced with half the subjects tested in the morning and half the subjects tested in the afternoon. Testers were blind as to what the subject’s I.Q. or WRAT or other inclusion criteria at the time of assignment to morning or afternoon test times. All subjects were given an eight-item “laterality” test consisting of three tasks to determine eye dominance, two tasks to determine foot dominance, and three tasks to determine hand dominance. Scores ranged from −8 (representing strong sinistral preference or left handedness), to + 8 (representing strong dextral preference or right handedness). Dextral dominant children were defined as having a laterality score of ≥2 and sinistral dominant children were defined as having a laterality score of ≤−2. Only approximately 9% of the subjects had laterality scores ≤2 and 87% of the subjects had laterality scores ≥2 and thus the majority of subjects in this study were right side dominant.

As shown in Table 1, age was not a confounding variable because there were no statistically significant differences in age between different I.Q. groups (low I.Q. vs hi I.Q. t = 1.949, df = 1/148, P = 0.06; low I.Q. vs middle I.Q, t = 1.787, df = 1/290, P = 0.076; hi I.Q, vs middle I.Q. t = 1.821, df = 1/298, P = 0.073). Gender was 55.6% male and 44.4% female and there were no significant differences in gender between the different I.Q. groups (t ranged from 0.059 to 0.295, P values ranged from 0.77 to 0.95). There was a significant difference in the socioeconomic status of the parents of the high I.Q. group vs the low I.Q. group (t = 5.65, P < 0.05) but not between the middle I.Q. group and the other two groups. The full scale I.Q. and age means, ranges and standard deviations of the subjects are shown in Table 1.

### Neuropsychological Measures

Neuropsychological and school achievement tests were administered on the same day that the EEG was recorded. The order of EEG and neuropsychological testing was randomized and counter-balanced so that EEG was measured before neuropsychological tests in one half the subjects and neuropsychological tests were administered before the EEG in the other half the subjects. The Wechler Intelligence Scale for Children revised (WISC-R) was administered for individuals between 5 years of age and 16 years and the Weschler Adult Intelligence Scale revised (WAIS-R) was administered to subjects older than 16 years. The neuropsychological sub-tests for estimating full scale I.Q. were the same for the WISC-R and the WAIS and included information, mathematics, vocabulary, block design, digit span, picture completion, coding and mazes.

### EEG Recording

Power spectral analyses were performed on 58 seconds to 2 minute 17 second segments of EEG recorded during resting eyes closed condition. The EEG was recorded from 19 scalp locations based on the International 10/20 system of electrode placement, using linked ears as a reference in the resting eyes closed condition. Subjects were instructed to close their eyes, relax and to try not to move their eyes during the recording. The trained EEG technicians were blind as to the subject’s I.Q. or WRAT and other inclusion criteria at the time of the EEG recording. The EEG was continually monitored during acquisition and if any electrodes were bad then the recording was paused and the electrode replaced. All subjects provided 19 channels of EEG plus a bipolar eye monitor channel. Eye movement electrodes were applied to monitor artifact and all EEG records were visually inspected and manually edited to remove any visible artifact. Each EEG record was plotted and visually examined and split-half reliability and test re-test reliability measures of the artifact free data were computed using the Neuroguide software program (NeuroGuide, v2.8.9). Split-half reliability tests were conducted on the edited artifact free EEG segments and only records with >90% reliability were entered into the spectral analyses. The amplifiers were designed and built by engineers at the NYU School of Medicine and amplifier bandwidths were nominally 1.0 to 30 Hz, the outputs being 3 db down at these frequencies. The EEG was digitized at 100 Hz and up-sampled to 128 Hz and then spectral analyzed using complex demodulation^23^,^24^,^25^.

### Power Spectral Analyses

A Fast Fourier transform (FFT) auto-spectral and cross-spectral analysis was computed on 2 second epochs thus yielding a 0.5 Hz frequency resolution over the frequency range from 0 to 30 Hz for each epoch. A ratio of the microvolt sine wave calibration signals from 0 to 30 Hz that were used to calibrate the University of Maryland amplifier frequency characteristics and the Lexicor NRS-24 amplifier characteristics were computed and then used as equilibration ratios in the FFT to exactly equate the two amplifier systems. The 75% sliding window method of Kaiser and Sterman^26^ was used to compute the FFT in which successive two-second epochs (i.e., 256 points) were overlapped by 500 millisecond steps (64 points) in order to minimize the effects of the FFT windowing procedure.

### Phase Slope Index

The Phase Slope Index (PSI) used the FFT to estimate the magnitude and the direction of the information flow between all 171 combinations of the 19 channel EEG data. The EEG phase slope index (PSI) estimates the temporal order of two signals separated in space, which is then interpreted as a driver-responder relation^1^,^2^,^3^,^27^. The basic hypothesis relies on the phase linearity between signals. PSI is based on the slope of the phase of the cross-spectrum between two time series. The idea is to define an average measure in such a way that this quantity properly represents relative time delays between signals separated in space. The PSI is computed as:


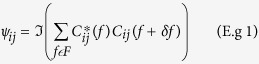


where ψ_ij_ is the coherency function between channel *i* and *j, δf* is the frequency resolution in our case = 0.5 Hz bins and 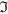 denotes the imaginary part of coherency. The coherency is defined as:


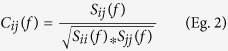


where:


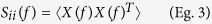



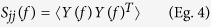



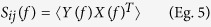


This calculation is made for each frequency bin and then we calculate the slope of the phase differences between scalp electrode locations for specific frequency bands, i.e., delta (1–4 Hz); theta (4–8 Hz); alpha frequency band (8.0–13 Hz); alpha1 (8–10 Hz); alpha2 (10–13 Hz); beta1 (13–15 Hz); beta2 (15–18 Hz); beta3 (18–25 Hz) and hi-beta (25–30 Hz). If the imaginary part of coherency between any channel pair approximates zero then this may indicate volume conduction. If the imaginary part is greater than zero or ψ_*ij*_ > 0 then the signal *i* happened before the signal *j* and *i* is the driver and *j* is the responder. If the slope of the imaginary part is negative or less than zero or ψ_*ij*_ > 0 then the signal *j* happened before the signal *i* and *j* is the driver and *i* is the responder.

### Selection of Variables for Discriminant Analyses Between High and Low I.Q. groups

The subjects were separated into a high full scale IQ group (I.Q. ≥120) and a low full scale I.Q. group (≤90 I.Q.) for purposes of the full scale I.Q. analyses In order to assess possible confounding by age, t-tests were conducted of differences between age in different I.Q. groupings (low I.Q. vs. middle I.Q., low I.Q. vs. high I.Q. and middle I.Q. vs. high I.Q.). The results of the analysis showed that there were no statistically significant differences in age between any of the I.Q. groupings.

There were 171 scalp electrode combinations and eight frequencies for a total 1,368 absolute PSI variables. T-tests between the high I.Q and low I.Q. group were conducted on all 1,368 EEG phase slope index (PSI) measures and a total of 124 variables were statistically significant at P < 0.05 were identified. PSI variables that were statistically significant at P < 0.05, were then entered into a step-wise discriminant analysis. The step-wise discriminant analysis selected a total of 42 variables representing a data reduction of 97% (e.g., 42/1,368) and a subject to variable ratio of 3.57. The delta and theta frequency bands contributed only 9% of the variables and the Alpha and Beta frequency bands contributed 91% of the variables in the discriminant function. Sensitivity, specificity, positive predicted values (PPV) and negative predicted values (NPV) were defined as: Sensitivity = True positives (TP)/(TP + False Negatives (FN)). Specificity was defined as: True Negatives (TN)/(TN + False Positives (FP)). PPV = TP/(TP + FP) and NPV = TN/(FN + TN).

## Results

### Discriminant Analysis of High I.Q. vs Low I.Q. groups

Table 2 is a summary of the number of subjects and classification accuracy of the discriminant analyses showing a discriminant classification accuracy of 99%. The sensitivity = 97.3% and specificity = 100%. The positive predicted value (PPV) = 100% and negative predicted value (NPV) = 97.5%. An independent cross-validation test was for the intermediate I.Q. group (90< and <120). As shown in Table 2, the independent cross-validation is where the intermediate I.Q. group was approximately evenly classified in the two extreme high vs low I.Q. groups which is expected if there is an approximate linear relationship between I.Q. and the phase slope index estimate of information flow.

In addition, as shown in Table 2 a leave-one-out (jackknife) cross-validation was conducted between the high and low I.Q. groups. The jackknife cross-validation yielded an overall classification accuracy = 94%, sensitivity = 94.3% and specificity = 93.8%. The positive predicted value (PPV) = 93.0% and negative predicted value (NPV) = 94.9%.

**Figure 1:**
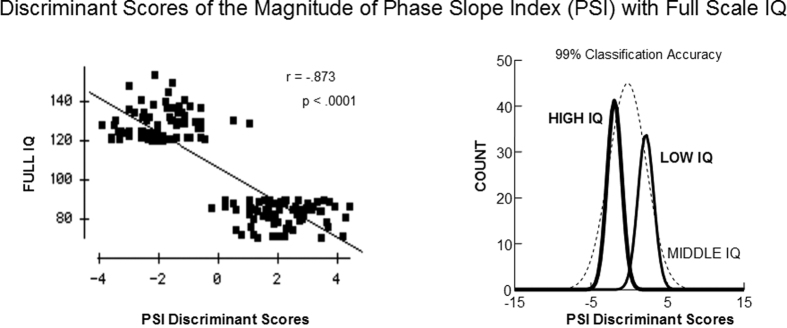
Results of discriminant analyses. Left is a scatter plot of discriminant scores of the high I.Q. and low I.Q. groups. Right is the distribution of discriminant scores for the high and low I.Q. groups as well as the intermediate I.Q. group. The intermediate subject’s distribution was midway between the high and low groups and serves as a cross-validation test. Also, the distribution supports a linear relationship between the magnitude of information flow and intelligence.

Figure 1 shows the distribution of the discriminant scores. The left is a scatter plot of discriminant scores of the high I.Q. and low I.Q. groups. Right is the distribution of discriminant scores for the high and low I.Q. groups as well as the intermediate I.Q. group. The intermediate subject’s distribution was midway between the high and low groups and serves as a cross-validation test. Also, the distribution supports a linear relationship between the magnitude of information flow and intelligence.

**Figure 2:**
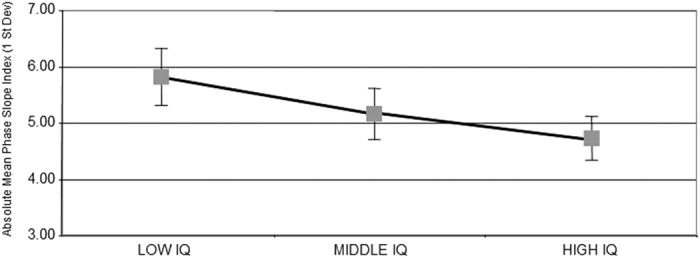
The mean absolute phase slope index of the 42 variables that were used in the discriminant function for the three I.Q. groups. Error bars = 1 st. dev. The results show that there was less information flow in the high I.Q. group in comparison to the other two groups. Also, the results show an inverse linear relationship between information flow and I.Q.

Figure 2 shows the mean values of the 42 absolute PSI variables that were used in the discriminant analyses for the three groups of I.Q. subjects. T tests were statistically significant between the high I.Q. and low I.Q. groups (t (df = 1, 149) = 3.176; P < 0.0001) and between the high I.Q. and intermediate I.Q. groups (t (df = 1, 301) = 1.837, P < 0.0001) and between the low I.Q and intermediate I.Q. groups (t (df = 1, 293) = 2.337; P < 0.0001). The lowest mean absolute PSI or information flow was in the high I.Q. group and the largest mean absolute PSI in the low I.Q. group with the intermediate I.Q. group midway between the high and low I.Q. groups. The results show an inverse linear relationship between information flow and I.Q.

### Inter-electrode Distance and Information Flow Short vs Long Distance

**Figure 3:**
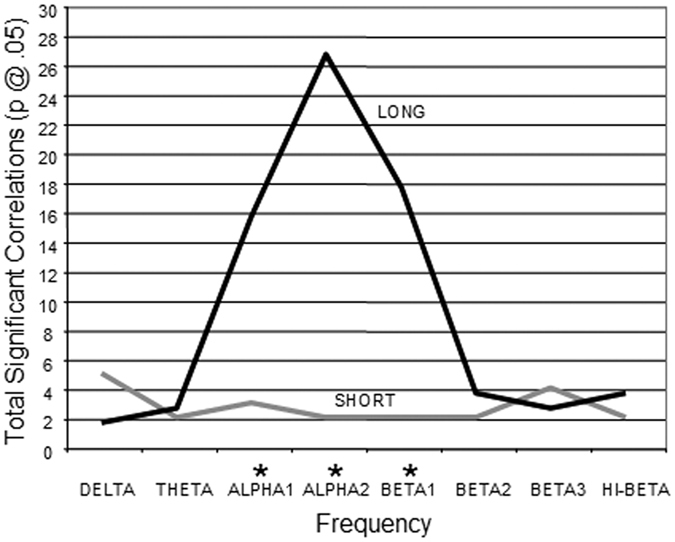
Line plot showing that the most significant correlations between I.Q. and magnitude of information flow is in the long distance inter-electrode combinations (i.e., 18 cm to 24 cm) in alpha and beta1 frequency bands. *Chi Square statistically significant.

Figure 3 is a bar graph showing that the most significant correlations between I.Q. and magnitude of information flow is in the long distance inter-electrode combinations in the Alpah1 and Alpha2 frequency bands. Statistically significant differences between short (6 cm to 12 cm) vs long (18 cm to 24 cm) inter-electrode distances using a Chi Square statistical test were for Alpha1 (X^2^ = 29.79, P < 0.0001) and Alpha2 (X^2^ = 34.55, P < 0.0001).

### Left vs Right Hemisphere

**Figure 4:**
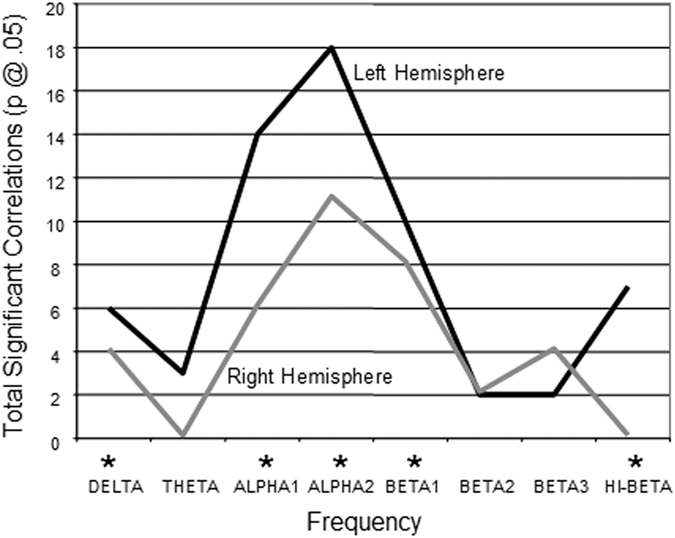
Line plot of the total number of statistically significant correlations (P < 0.05) between PSI and I.Q. in left and right hemispheres. There was a significant hemispheric difference with the left hemisphere exhibiting higher correlations between information flow and I.Q. than the right hemisphere. Also, Alpha1 and Alpha2 frequency bands were the most significant. *Chi Square statistically significant.

Figure 4 is a line graph showing that there is a significant hemispheric difference with the left hemisphere exhibiting higher correlations between information flow and I.Q. than the right hemisphere. Chi Square statistical tests for Delta (X^2^ = 17.26, P < 0.0001) Alpha1 (X^2^ = 101.79, P < 0.00001), Alpha2 (X^2^ = 238.09, P < 0.00001), Beta1 (X^2^ = 79.25, P < 0.00001) and Hi-Beta (X^2^ = 5.64, P < 0.0176).

### Frontal and Parietal Phase Slope Index and Intelligence

**Figure 5:**
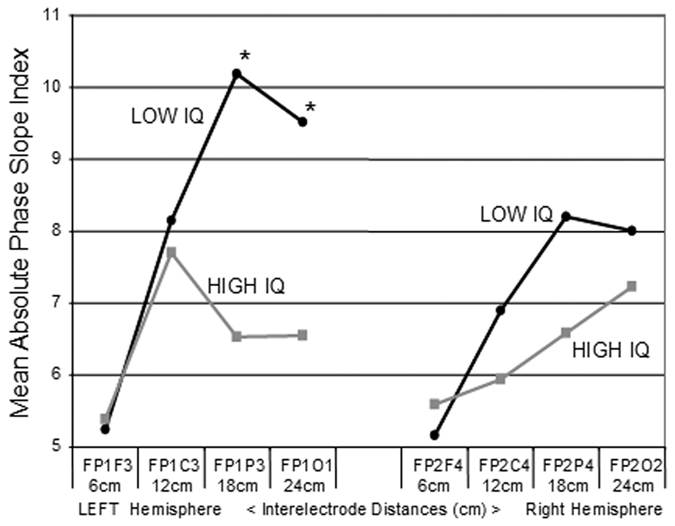
Mean absolute phase slope index in the left and right frontal electrode combinations as a function of distance from the Fp1 and Fp2 electrodes for the low I.Q. (gray square) and high I.Q. (black dot) groups in the Alpha2 frequency band. The electrode combinations were Fp1/2-F3/4, Fp1/2-C3/4, Fp1/2-P3/4 and Fp1/2-O1/2. The values on the left are from the left hemisphere and the values on the right are from the right hemisphere. * T-tests of mean differences between low vs high I.Q. groups were statistically significant at Fp1-P3 (t (df = 149) = 3.071, P < 0.0025) and at Fp1-O1 (t (df = 149) = 2.328, P < 0.0213).

Figure 5 shows the mean absolute PSI for different distances between electrode pairs in the anterior to posterior direction for the left and right hemispheres in the Alpha2 frequency band. The largest differences in mean absolute PSI between the high I.Q. and low I.Q. groups were between the frontal and parietal (Fp1/P3 and Fp2/P4) regions. In the left hemisphere t-tests were significant for the Fp1-P3 (t(149) = 3.071, P < 0.0025) and Fp1-O1 (t (149) = 2.328, P < 0.0213) electrode combinations. None of the differences in mean phase slope index were statistically significant although the Fp2-P4 had the largest difference in means.

## Discussion

This study extends the investigation of the relationship between EEG connectivity measures and intelligence by showing significant correlations between intelligence and estimates of information flow using the phase slope index. While information flow was present in all subjects, a linear inverse relationship was demonstrated in which the higher I.Q. then the less the magnitude of information flow between EEG scalp locations as measured by the phase slope index^1^,^2^,^3^. Also, the largest difference in information flow between the high and low I.Q. groups was in the frontal-parietal electrode combinations in the alpha frequency bands. This finding is consistent with EEG coherence and phase measures of intelligence published previously^10^.

Another finding was that differences in information flow between high and low I.Q. groups were primarily in long distance inter-electrode combinations. This finding is opposite to the relationship between I.Q. scores and EEG phase reset in which short inter-electrode distances (e.g., 6 cm to 12 cm) were more strongly correlated with intelligence than the long inter-electrode distances^11^. Also, the left hemisphere exhibited stronger correlations between magnitude of information flow and intelligence in comparison to the right hemisphere. A stronger left hemisphere relationship to I.Q. was also observed in correlations between phase reset and intelligence^11^. The finding of stronger left hemisphere effects is likely due to the fact that the WISC-R intelligence test is largely a verbal test of intelligence.

In summary the results of this study support both of the most common theories where intelligence is related to: 1- specialized frontal-parietal connectivity and, 2- general efficiency of information processing.

### Intelligence, Efficiency and Homeostatic Neuroplasticity

The finding of reduced magnitude of information flow in higher I.Q. subjects in long distance inter-electrode combinations is best interpreted in the context of other network correlations with intelligence. For example, correlations between phase shift and phase lock duration were statistically significant in short inter-electrode combinations that reflect information processing in local or segregated clusters of neurons^11^. The longer the phase shift duration then the higher the I.Q. where phase shift duration was interpreted as a recruiting process to synchronize available neurons at a given moment of time^11^,^28^. The current study when also considering the phase reset relations to intelligence indicates that the lower magnitude of information flow in high I.Q. subjects represents a more efficient local information processing where there is reduced demand for neural resources located in distant clusters of neurons. Information flow occurs in all subjects, however, the magnitude of information flow between brain regions is less in the higher I.Q. subjects as seen in Fig. 3. This indicates that each network hub receives and sends information to all other network hubs but if a given hub has inefficient information processing in the local domain then compensatory hubs send information to the weak hubs in order to achieve maximum efficiency of information processing in the network as a whole.

This is consistent with a homeostatic neuroplasticity model of intelligence in which maintenance of an optimal small-world dynamic involves minimizing long distance information processing and maximizing the efficiency of local information processing^29^. Phase reset operates primarily in the local hub domain to recruit and allocate resources to efficiently process information while information flow operates in the long range compartments to compensate for inefficiencies in the local domain. The greater the small-world efficiency of the global brain networks then the higher is performance on the WISC-R I.Q. intelligence test. Graph theoretical models of intelligence using structural MRI^16^,^17^ found that higher I.Q. is negatively correlated with path length and path length is inversely proportional to network efficiency. All three types of connectivity, that is, structural connectivity, functional connectivity and effective connectivity demonstrate a positive correlation between I.Q. and the efficiency of information processing in networks of the brain. Long distance information flow and local phase reset are part of the underlying dynamics in which neural resources are quickly identified and allocated in local functional clusters or hubs embedded in a small-world network with high speed homeostatic plasticity to maintain function even when there is loss of neurons (high resiliency).

### A Small-World Model of Intelligence

**Figure 6:**
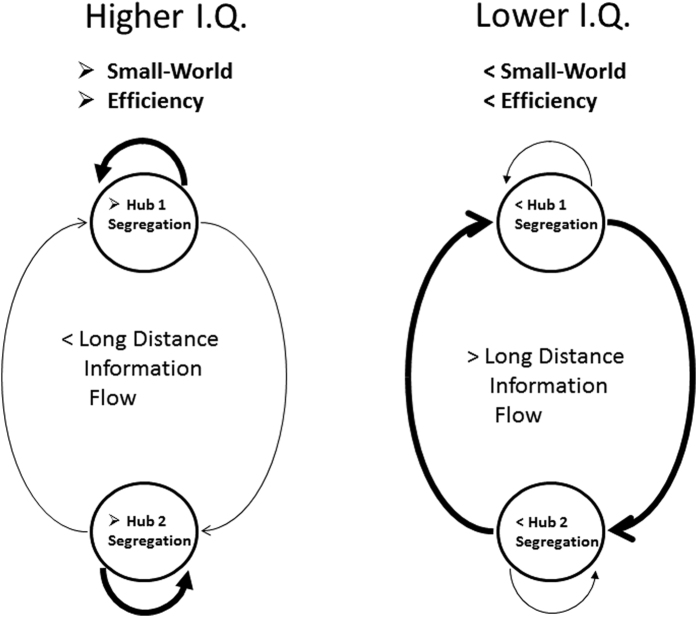
An illustration of the relationships between network connectivity and intelligence with special emphasis on the small-world model. The thickness of the lines reflect the differences in local hub vs long distance information processing. The small-world model minimizes long distance expenses and maximizes local information processing. Higher I.Q. subjects have longer phase shift durations and shorter phase lock durations in local connections resulting in the efficient recruitment of large numbers of neurons for short periods of time. Because of local processing efficiency high I.Q. subjects have lower information demands placed on other hubs of the network and thus there is lower information flow in the long distance system. Lower I.Q. subjects appear to have less efficient information processing in local regions of the brain which places higher demand on the long distance connections linking hubs of the network. Homeostatic neuroplasticity dynamically allocates resources and compensates for inefficiencies in the segregated and integrated hubs of the network at each instant of time.

Complexity in the brain is often defined by models of information theory and stochastic processes involving a balance between differentiation and integration^30^,^31^,^32^. Tononi *et al*.^30^ showed that highly complex neural networks were characterized by neurons that were organized into densely linked groups that were sparsely and reciprocally interconnected. Small-world models of connected systems show that reduction of long distance connections and increased connectivity of local systems is a fundamental information optimization process^33^,^34^,^35^. Figure 6 is an illustration of the network efficiency differences between the high vs low I.Q. groups. Small-world models of the brain emphasize the tradeoffs between neuron size, conduction velocity, noise and energy that structures the operation of the brain so that it operates with large spatial clusters of neurons synchronized in discrete temporal frames^28^,^36^,^37^. A reasonable model is that local efficiency of information processing is reflected by the balanced of chaos and stability in local clusters of neurons as measured by long phase shift durations and short phase lock durations^11^,^18^ resulting in reduced demands on the more biologically expensive long distance connections. Also, efficiency and intelligence are related by virtue of specialized frontal-parietal information processing and efficient allocation of resources by two way connected clusters of neurons or in Graph theory high functional cluster coefficients and short path lengths.

### Intelligence and Frontal and Parietal Lobes

Previous studies from this laboratory reported an inverse relationship between phase differences and I.Q^9^. and also phase lock durations were shorter in higher I.Q. subjects in bilateral frontal and parietal relations^11^. Nunez^38^ estimated that approximately 50% of the amplitude of the EEG arises directly beneath a given scalp electrode and 95% is contributed by sources up to 6 cm distant. Therefore, caution should be exercised in relating EEG sources to a scalp location, nonetheless, the fact that the electrodes located over the parietal and frontal regions indicates that one cannot preclude parietal and frontal contributions. The findings in this study on effective connectivity are consistent with these previous studies but add an important new understanding of the efficiency of two-way flows of information between frontal and parietal regions and intelligence. The findings in this study indicate the more efficient local information processing is in frontal and parietal regions then the higher the I.Q. because of reduced demand on the long distance connections. Significant information flow is present between the frontal and parietal regions in all subjects, however, homeostatic balance and high speed allocation of resources is optimal in the higher I.Q. subjects. The frontal-parietal lobes are involved in multiple aspects of attention and especially orienting and executive aspects of attention^39^,^40^,^41^. Intelligence is fundamentally linked to attention because of the importance of stimulus selection as well as verbal and spatial information in a system with limited capacity to simultaneously process information. Focused attention is a critical capacity mediated by frontal-parietal networks as the entry to the conscious state necessary to produce the global work space associated with consciousness^42^. This literature and the findings in this study supports a dual-dynamic underlying intelligence: 1- Specialized frontal and parietal lobe networks and, 2- Efficient local information processing is related to reduced demand on long distance connections and increased global efficiency.

## Additional Information

**How to cite this article**: Thatcher, R. W. *et al*. Intelligence and eeg measures of information flow: efficiency and homeostatic neuroplasticity. *Sci. Rep.* **6**, 38890; doi: 10.1038/srep38890 (2016).

**Publisher's note:** Springer Nature remains neutral with regard to jurisdictional claims in published maps and institutional affiliations.

## Figures and Tables

**Table 1:** Group sample sizes and age and I.Q. Descriptive Statistics.

IQ Groups	N	Mean age	SD age	Age range	Mean full IQ	SD full IQ	Full IQ Range
Low IQ	71	11.31	3.08	5.02–17.18	82.65	6.12	70–90
Middle IQ	221	10.46	3.26	5.00–17.54	105.48	7.54	91–119
High IQ	79	9.50	2.85	5.14–15.80	128.52	7.68	120–154

The three I.Q. groups were selected solely based on the range of the full-scale I.Q. scores as shown in the column to the right in Table 1.

**Table 2:** Discrimintant Analysis and Jackknife Replication.

IQ GROUP	N	IQ< = 90	IQ> = 120
Classification Accuracy = 99%			
Full IQ< = 90	n = 71	71 (100%)	0 (0%)
Full IQ> = 120	n = 79	2 (3%)	77 (97%)
90< Full IQ <120	n = 221	100 (45%)	121 (55%)
Jackknifed Classification Accuracy = 94%			
IQ GROUP	N	IQ< = 90	IQ> = 120
Full IQ < = 90	n = 71	66 (93%)	5 (7%)
Full IQ> = 120	n = 79	4 (3%)	75 (95%)
